# *Streptococcus mitis* Expressing Pneumococcal Serotype 1 Capsule

**DOI:** 10.1038/s41598-018-35921-3

**Published:** 2018-12-19

**Authors:** Fernanda C. Lessa, Jennifer Milucky, Nadine G. Rouphael, Nancy M. Bennett, H. Keipp Talbot, Lee H. Harrison, Monica M. Farley, Jeremy Walston, Fabiana Pimenta, Robert E. Gertz, Gowrisankar Rajam, Maria da Gloria Carvalho, Bernard Beall, Cynthia G. Whitney

**Affiliations:** 1 Centers for Disease Control and Prevention, National Center for Immunization and Respiratory Diseases, Division of Bacterial Diseases, Atlanta, Georgia USA; 2 Emory University School of Medicine, Department of Medicine, Atlanta, Georgia USA; 3 University of Rochester School of Medicine and Dentistry, Department of Medicine, Rochester, New York USA; 4 Vanderbilt University Medical Center, Nashville, Tennessee USA; 5 Johns Hopkins Bloomberg School of Public Health, Baltimore, Maryland USA; 6 Atlanta Veterans Affairs Medical Center, Atlanta, Georgia USA; 7 Division of Geriatric Medicine and Gerontology, Johns Hopkins University School of Medicine, Baltimore, Maryland USA

## Abstract

*Streptococcus pneumoniae’s* polysaccharide capsule is an important virulence factor; vaccine-induced immunity to specific capsular polysaccharide effectively prevents disease. Serotype 1 *S*. *pneumoniae* is rarely found in healthy persons, but is highly invasive and a common cause of meningitis outbreaks and invasive disease outside of the United States. Here we show that genes for polysaccharide capsule similar to those expressed by pneumococci were commonly detected by polymerase chain reaction among upper respiratory tract samples from older US adults not carrying pneumococci. Serotype 1-specific genes were predominantly detected. In five oropharyngeal samples tested, serotype 1 gene belonging to *S*. *mitis* expressed capsules immunologically indistinct from pneumococcal capsules. Whole genome sequencing revealed three distinct *S*. *mitis* clones, each representing a *cps1* operon highly similar to the pneumococcal *cps1* reference operon. These findings raise important questions about the contribution of commensal streptococci to natural immunity against pneumococci, a leading cause of mortality worldwide.

## Introduction

*Streptococcus pneumoniae*’*s* polysaccharide capsule is believed to be its most important virulence factor, and natural or vaccine-induced immunity to the capsule is protective against disease. The capsular polysaccharide structure is highly variable, with over 90 different pneumococcal serotypes identified. In the early 20^th^ century, serotype 1 was the most commonly identified serotype causing pneumococcal pneumonia and sepsis in the United States^1^. However, over the last few decades and well before the introduction of a 13-valent pneumococcal conjugate vaccine (PCV13) targeting serotype 1 into the national infant immunization program in 2010, serotype 1 became a rare cause of pneumococcal disease in the US population, with only sporadic increases being reported in Utah^2,3^. Among 42,059 cases of invasive pneumococcal disease (IPD) identified through Active Bacterial Core surveillance (ABCs), part of the Centers for Disease Control and Prevention’s Emerging Infections Program, from 1998 through 2009 (before PCV13 introduction), 609 (1.4%) were due to serotype 1 (CDC unpublished data).

At the same time, serotype 1 is a common cause of severe disease in several countries outside the United States. In African countries, serotype 1 remains a leading cause of IPD with the potential to cause large outbreaks and epidemics, even in countries that have already introduced PCV13, as recently reported in Ghana^4–8^. In England and Wales, serotype 1 was among the most common serotypes causing invasive disease in cases reported from 2008 to 2014^9^. Serotype 1 pneumococci are significantly more resistant to opsonization and complement deposition compared to some other serotypes, which could explain the high degree of invasiveness of this serotype and its potential to cause epidemics^10^. Conversely, serotype 1 pneumococci rarely cause asymptomatic colonization of the upper respiratory tract in children or adults^11,12^. The reason that pneumococcal serotype 1 disease is now unusual in the United States but continues to commonly cause disease elsewhere is not understood; whether natural exposure to the serotype 1 capsule varies between populations is unknown.

In this report, we describe commensal streptococci – *Streptococcus mitis* – expressing pneumococcal serotype 1 capsule, which were discovered during a 2015–2016 study of pneumococcal colonization in US adults aged 65 years and older. Our results suggest that serotype 1-containing *S*. *mitis* may be common among US adults. In addition, we describe results of tests of genetic similarity and serologic cross-reactivity with serotype 1 pneumococci.

## Methods

### Study Design and Population

We conducted a cross-sectional survey from July 13, 2015 through March 31, 2016 among healthy adults 65 years of age and older enrolled from outpatient clinics, senior residential communities, senior day care centers and health fairs in four US states participating in the Centers for Disease Control and Prevention’s (CDC) Active Bacterial Core surveillance: Georgia, Maryland, New York and Tennessee. We excluded adults if they reported severe immunocompromising conditions (e.g., hematologic malignancies, transplant recipient, end-stage renal disease)^13^ or if they were residents in nursing homes or skilled nursing facilities.

For each eligible adult who consented to participate, we collected one nasopharyngeal (NP) and one oropharyngeal (OP) swab and conducted an interview to obtain demographic, clinical and vaccination data. Participants without paired NP and OP specimens were excluded from the study. Trained staff followed up with healthcare providers to confirm PCV13 receipt and dates of immunization.

The study was approved by the institutional review boards of the Centers for Disease Control and Prevention (CDC – Protocol #6725), Emory University, Georgia Department of Public Health, Maryland Department of Health, Johns Hopkins School of Public Health, University of Rochester, Vanderbilt University, and Tennessee Department of Health. Written informed consent was obtained from all study participants, and the research was performed according to federal regulations for protection of human subjects.

### Specimen collection and processing

Trained staff collected NP and OP specimens using sterile nylon tipped flocked swabs (Copan Diagnostics INC, Murrieta, CA). NP and OP specimens were placed separately into cryovials containing 1.0 ml skim milk-tryptone-glucose-glycerol (STGG) medium, vortexed for 10 to 20 seconds to disperse the organisms from the swab, and frozen at −70 °C within 4 hours of collection.

Each swab was processed separately. For pneumococcal isolation and identification, 200 µl of either STGG-NP or STGG-OP inoculated medium was transferred into 5.0 ml Todd Hewitt broth containing 0.5% yeast extract (THY) and 1 ml of rabbit serum, which was then incubated at 37 °C in a CO_2_ incubator for 5–6 hours. After incubation, 10 µl of cultured broth was streaked on tryptone soy agar plates with 5% sheep blood (BAP) and incubated at 37 °C in CO_2_ for 18–24 hours^14^. Alpha-hemolytic colonies resembling pneumococci were tested for susceptibility to optochin and bile solubility, and serotyped by Quellung using CDC pneumococcal typing antisera^15^. Pneumococcal culture-negative NP and OP specimens underwent real-time PCR targeting *lytA* for detection of pneumococcal DNA^16^. DNA extraction was performed using a MagNA Pure Compact with an external lysis protocol using buffer #4 (Roche isolation kit III) or NucliSENS® EasyMag® automated system for total nucleic acid extraction, according to manufacture instructions. Specimens were considered to be *lytA*-positive if the cycle threshold (Ct) value was ≤35. Real-time multiplex PCR for detection of 37 serotypes (including PCV13 serotypes) was performed on culture-negative specimens from 53 and 395 adults with *lytA*-positive and *lytA*-negative specimens, respectively. For *lytA*-negative specimens, we assumed that 30% (+/−5%) would have a serotype detected by real-time PCR leading to a target sample size of 395; while for *lytA*-*positive* specimens almost all of them (71%) were tested. PCR for serotyping was performed using DNA extracted from 200 µl of each NP and OP swab-inoculated STGG media^17^. Because real-time PCR for serotyping is a multiplex reaction, Ct ≤35 for a specific serotype was considered positive, while Ct values between 36 and 40 were considered equivocal and repeated three times for confirmation.

### Non-pneumococcal species isolation and characterization

The five specimens with the lowest PCR cycle threshold for serotype 1-specific *wzy1* gene, which lies within the pneumococcal *cps1* operon, were cultured using broth enrichment followed by plating on blood agar for isolation of alpha-hemolytic streptococcal species; these five were all OP specimens, two *lytA* positive and three *lytA* negative. Non-pneumococcal alpha-hemolytic streptococci colonies grown on the plates that were optochin resistant and bile insoluble were screened for *wzy1* by real-time and conventional multiplex PCR. *wzy1*-positive non-pneumococcal isolates (N = 5, 1 from each specimen) were subjected to *lytA* screening by PCR, immunochromatographic testing (BinaxNow), and whole genome sequencing on Illumina MiSeq^18^. We compared the sequences of the 16,000 bp non-pneumococcal streptococci *cps1* operons and flanking regions with the corresponding sequence of the 22,000 bp pneumococcal serotype 1 reference strain (GenBank accession CR931632) using the SSEARCH program^19^.

Species assignment was done by phylogenetic analysis of seven previously described concatenated housekeeping gene sequences (total length of 3063 bases)^20^. A selection of 3063 bp sequences^20^ that most closely matched the non-pneumococcal streptococcal strains were used to construct the phylogenetic tree. In addition, reference strain sequences were obtained from NCBI accessions for *S*. *pseudopneumoniae* (ATCCBAA960), *S*. *mitis* (B6 and NCTC 12261), *S*. *infantis* (ATCC 700779), and *S*. *oralis* (Uo5). Evolutionary distances were computed using the Maximum Composite Likelihood method^21^ and analyzed in MEGA7^22^.

### Capsule expression of non-pneumococcal streptococci isolates

Latex agglutination followed by the Quellung reaction using polyclonal serotype 1 *S*. *pneumoniae* typing antiserum prepared in rabbits was performed on the five isolates to assess capsule expression and compare capsule expression with that of *S*. *pneumoniae* serotype 1 (positive control). In addition, double immunodiffusion assays using the same antiserum assessed cross-reactivity of non-pneumococcal streptococci with serotype 1 pneumococcal antisera using the methods described by Sorensen *et al*.^23^. Briefly, both untreated (i.e. crude) bacterial suspension extracts and extracts treated with proteinase K were tested. The double immunodiffusion immunoprecipitation reaction was carried out on Pierce standard Ouchterlony agarose gel plates (Pierce #31111, Thermofisher Scientific) as previously described^24,25^. We applied 15 µl undiluted serotype 1 pneumococcal antiserum (AS) to the center well (2.8 mm) with 15 µl extract samples applied to the four surrounding wells, placed at a distance, edge to edge, of 3.2 mm. Two wells were left empty as controls. Plates were kept for 2 days at 5 °C and then photographed.

### Opsonophagocytosis of pneumococcal and non-pneumococcal streptococci isolates

An opsonophagocytic killing assay^26^ was used to assess functional antibody activity against both the non-pneumococcal streptococci strains expressing serotype 1 capsular polysaccharide and the serotype 1 pneumococcus. *S*. *mitis* ATCC (NCTC 12261) strain (PCR negative for *wzy1* gene) was used as a control. In a 96-well micro titer plate, 20 μl of *S*. *mitis* serotype 1 or serotype 1 pneumococcus was pre-opsonized with 10 μl anti-pneumococcal serotype 1 specific human polyclonal serum (8-dilutions, diluted 2-fold starting neat) or anti-*S*. *mitis* serotype 1 rabbit serum for 15 minutes at 37 °C with 5% CO_2_. After pre-opsonization, 10 μl baby rabbit complement was added followed by 40 μl of human promyelocyte (HL60) derived neutrophils (effector cells). Complement, neutrophil, and bacterial controls were maintained. After incubation for 45 minutes at 37 °C, 5 μl from each well was transferred onto air-dried Todd-Hewitt yeast extract agar. Opsonophagocytic titers were the reciprocal of the serum dilution with >50% killing compared with the growth in the complement control wells.

### Data Analysis

Analyses were performed using SAS software, version 9.3. We calculated the prevalence of pneumococcal carriage as determined by culture. Among culture-negative patients, we calculated the prevalence of serotype genes detected by PCR among patients with *lytA*-positive or *lytA*-negative specimens. Comparison of the prevalence of pneumococcal vaccine-serotype homolog genes (serotypes 1, 3, 4, 5, 6A, 6B, 7F, 9V, 14, 18C, 19A, 19F, 23F) by PCV13 status was done using the chi-square test. Patients were considered vaccinated if PCV13 was received at least 14 days before swab collection. Serotypes not distinguished by PCR (e.g. 9A/9V, 7A/7F, 18A/18B/18C/18F) were grouped.

The data that support the findings of this study are available from the corresponding authors upon reasonable request. The strains are stored in the CDC’s *Streptococcus* laboratory and can be available to investigators upon discussion and approval by the authors and site investigators. The method (composition of the strain with the purpose to induce immune response) is the subject of a U.S. Provisional Patent Application, No. 62/627,602 filed on February 7, 2018; non-exclusive royalty-free (NERF) academic research use licenses are available upon request.

### Disclaimer

The findings and conclusions in this report are those of the authors and do not necessarily represent the official position of the Centers for Disease Control and Prevention.

## Results

We enrolled 1,299 adults who had both NP and OP specimens successfully collected; of these, 67.9% were female, median age was 74 years (range: 65–102 years), and 73.9% were either recruited in Georgia or New York. Pneumococcus was isolated from specimens from 16 (1.2%) participants; 12 from NP, 2 from OP and 2 from both NP and OP. Of the 1,283 participants with culture-negative samples, 75 (5.8%) had a specimen that was *lytA*-positive: 1 from NP only, 71 from OP only, and 3 from both NP and OP (Supplementary Material, Figure).

PCR-based serotype deduction was performed on both NP and OP specimens from 448 culture-negative adults. Among the 53 adults with *lytA*-positive specimens from either NP or OP, 8 (15.1%) had *wzy1* detected in one or both specimens; among the 395 adults with *lytA*-negative specimens from both NP and OP, 41 (10.4%) had *wzy1* detected. Of the 49 adults with *wzy1*-positive, culture-negative specimens, 42 (85.6%) had *wzy1* detected only in the OP specimen, 4 (8.2%) in both NP and OP specimens, and 3 (6.1%) in the NP specimen only; 23 (46.9%) of these 49 were enrolled in Georgia, 11 (22.5%) in Tennessee, 10 (20.4%) in New York and five (10.2%) in Maryland.

Of the 53 and 395 adults with *lytA-*positive and *lytA*-negative specimens, 27(50.9%) and 131(33.2%) were positive by PCR for genes for other pneumococcal serotypes; although overall *wzy1* was most commonly detected (Table 1). Compared to NP specimens, OP specimens were more likely to be PCR-positive for serotype-specific pneumococcal genes in adults with *lytA*-positive (50.9% [27/53] vs. 3.8% [2/53]; P < 0.001) or *lytA*-negative (32.7% [129/395] vs. 2.3% [9/395]; P < 0.001) samples. From the 488 culture-negative adults with samples that underwent PCR for serotype genes, 424 had documented vaccination history and 251 received PCV13. Adults who were vaccinated with PCV13 were less likely to have pneumococci vaccine-type genes detected in their specimens compared to those who were unvaccinated (26.0% [45/173] vs. 35.8% [90/251]; P = 0.03).

**Table 1:** PCR serotyping results from pneumococcal culture-negative specimens by *lytA* status, USA, 2015–2016.

PCR Serotyping Results*	*lytA* positive specimens	*lytA* negative specimens
	(N = 53)	(N = 395)
	n (%)	n (%)
001	8 (15.1)	41 (10.4)
004	9 (16.9)	37 (9.4)
9V/9A^#^	6 (11.3)	32 (8.1)
005	1 (1.9)	11 (2.8)
18A/18B/18C/18F^#^	7 (13.2)	25 (6.3)
23A	2 (3.8)	4 (1.0)
19F	1 (1.9)	0 (0)
14	0	2 (0.5)
12F/12A/12B/44/46^#^	1 (1.9)	9 (2.3)
Others	2 (3.8)	13 (3.3)
Negative for the serotypes tested by PCR	26 (49.1)	264 (66.8)

Specimens were nasopharyngeal and oropharyngeal samples from 448 adults aged ≥65 years; those from adults with lytA-positive PCR results from either specimen were classified as *lytA* positive; those with both specimens testing *lytA* negative were classified as *lytA* negative.

*Not mutually exclusive.

^*#*^PCR reaction groups these serotypes.

### Isolation of non-pneumococcal strains and characterization

*Streptococcus mitis* was identified from culture from all 5 of the OP specimens, three *lytA*-negative (ID# L006, L115, L116) and two *lytA*-positive (ID# L121, L164), with the lowest cycle threshold values for *wzy1* (between 22.0 and 27.5). The *S*. *mitis* isolates were positive for the serotype 1-specific *wzy1* gene by both real-time and conventional PCR, and by latex agglutination for serotyping (Table 2). Two *S*. *mitis* isolates were Binax positive.

**Table 2:** Results of characterization analyses for *Streptoccocus mitis* strains.

Isolate ID	Type of Sample	lytA of OP specimen	Species isolated	Optochin	*lytA* of *S*. *mitis* isolate	Immunochro-matographic testing (BinaxNow)	Serotyping assays			*Capsule visualization by Quellung using pneumococcal anti-serum*
							Real-time PCR	Conventional PCR	Latex agglutination	
L006	OP	Negative	*S*. *mitis*	Resistant	Negative	Negative	Serotype 1	Serotype 1	Serotype 1	Weak visualization of capsule with serotype 1 anti-serum
L115	OP	Negative	*S*. *mitis*	Resistant	Negative	Positive	Serotype 1	Serotype 1	Serotype 1 (weak reaction)	No visualization
L116	OP	Negative	*S*. *mitis*	Resistant	Negative	Positive	Serotype 1	Serotype 1	Serotype 1 (weak reaction)	No visualization
L121	OP	Positive (Ct = 31.19)	*S*. *mitis*	Resistant	Negative	Negative	Serotype 1	Serotype 1	Serotype 1	Weak visualization of capsule with serotype 1 anti-serum
L164	OP	Positive (Ct = 28.98)	*S*. *mitis*	Resistant	Negative	Negative	Serotype 1	Serotype 1	Serotype 1 (weak reaction)	Weak visualization of capsule with serotype 1 anti-serum

Ct = cycle threshold.

**Figure 1:**
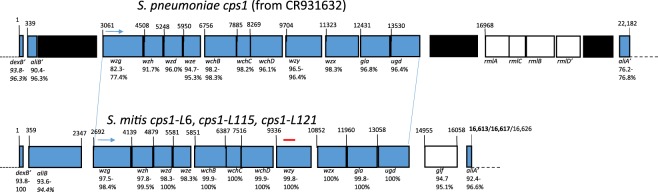
Genomic sequencing results showing alignment of *cps1* regions between serotype 1 *S*. *pneumoniae* and *S*. *mitis*. Comparison of *cps*1-L polysaccharide synthetic cluster regions from *S*. *mitis* strains L006/L164 (16,613 bp), L115/L116 (16,617 bp) and L121 (16,626 bp) with the corresponding pneumococcal *cps*1 sequence. White rectangles represent open reading frames not shared between the species. Top diagram depicts ranges of sequence identity between pneumococcal *cps1* genes and the *S*. *mitis* counterparts depicted on the bottom diagram. Ranges of sequence identity between the *S*. *mitis* alleles are given below *S*. *mitis cps1* diagram. L006 and L164 shared identical 16613 sequence. L115 and L116 shared identical 16617 bp sequence. The connecting blue lines depict the alignment of the polysaccharide synthesis clusters. Black regions in the pneumococcal diagram depict remnants of transposase genes. Two inactive pneumococcal genes (*aliB* and *rmlD*, the latter renders the rhamnose biosynthesis cluster inactive) containing frameshift mutations are indicated, as well as the partial 5′ and 3′ ends of the *dexB* and *aliA* genes in both species (′). The region detected through use of PCR assays to first detect the presence of these strains in upper respiratory specimens is depicted by the red line above the *wzy* gene.

Whole genome sequence analysis of these five *S*. *mitis* isolates revealed three very similar, although distinct, capsule gene operons and adjacent genes. The lengths of the three loci, corresponding to the region between the flanking *dexB* and *aliA* segments of the 22,182 bp pneumococcal reference (GenBank accession CR931632), ranged between 16,613–16,626 bp and shared 98.0–98.2% sequence identity with one another. Each of the 11 genes within the polysaccharide synthetic cluster (*wzg – ugd*) were highly homologous to the *S*. *pneumoniae* serotype1 counterparts and shared the same order (Fig. 1).

**Figure 2:**
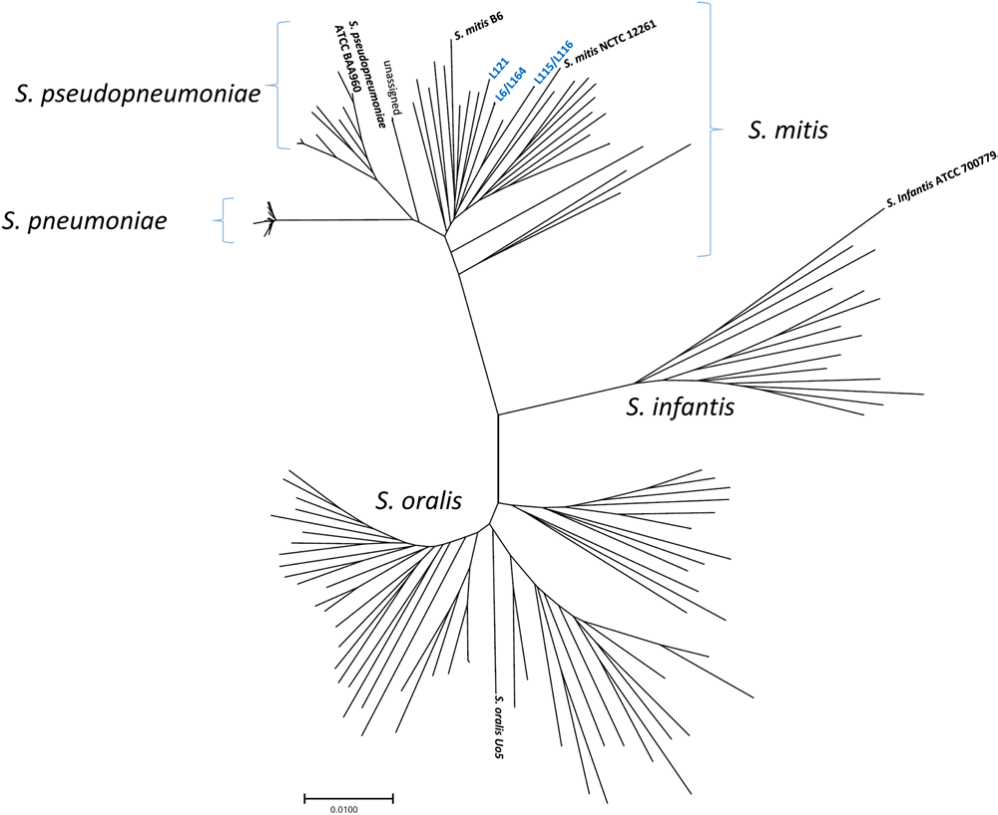
Species assignment of five *Streptococcus mitis* strains based upon phylogeny of concatenated housekeeping gene segments. Tree showing the positions of well-characterized strains described by Bishop *et al*.^20^ and the newly-identify *S*. *mitis* strains with pneumococcal *cps1* homologues, shown in blue. Evolutionary distances were analyzed using MEGA 7^22^.

Three distinct *S*. *mitis* clones were found (Fig. 2). While 16 different pneumococcal strains representing 6 different serotypes formed a tight cluster based upon phylogenetic analysis of 3,063 bp concatenated housekeeping gene segments, the *S*. *mitis* strains formed a much more divergent cluster. At the level of direct DNA sequence comparison, the 3 serotype 1 *S*. *mitis* clones shared 97.1–97.3% sequence identity over the 3,063 bp, while the 16 pneumococcal strains shared 99.5–99.9% sequence identity. These *S*. *mitis* strains were all macrolide-resistant (all contained *erm*-methylase or *mef* genes) and cotrimoxazole-resistant (alterations within *folA* and/or *folP* genes). As in pneumococci, the *cps* loci in the *S*. *mitis* strains were situated between the penicillin binding protein genes *pbp2x* and *pbp1a*. All five *S*. *mitis* strains lacked the pneumococcal-specific target *piaA* but contained pneumolysin genes with 61–97% sequence identity to the pneumococcal *ply* gene; suggesting that *piaA* is pneumococcal-specific, whereas *ply* may be found within *S*. *mitis* strains with varying degrees of similarity with pneumococci.

### Cross-reactivity between *S*. *mitis* and serotype 1 *S*. *pneumoniae* anti-serum and between *S*. *pneumoniae* serotype 1 and anti-*S*. *mitis* serotype 1 anti-serum

**Figure 3:**
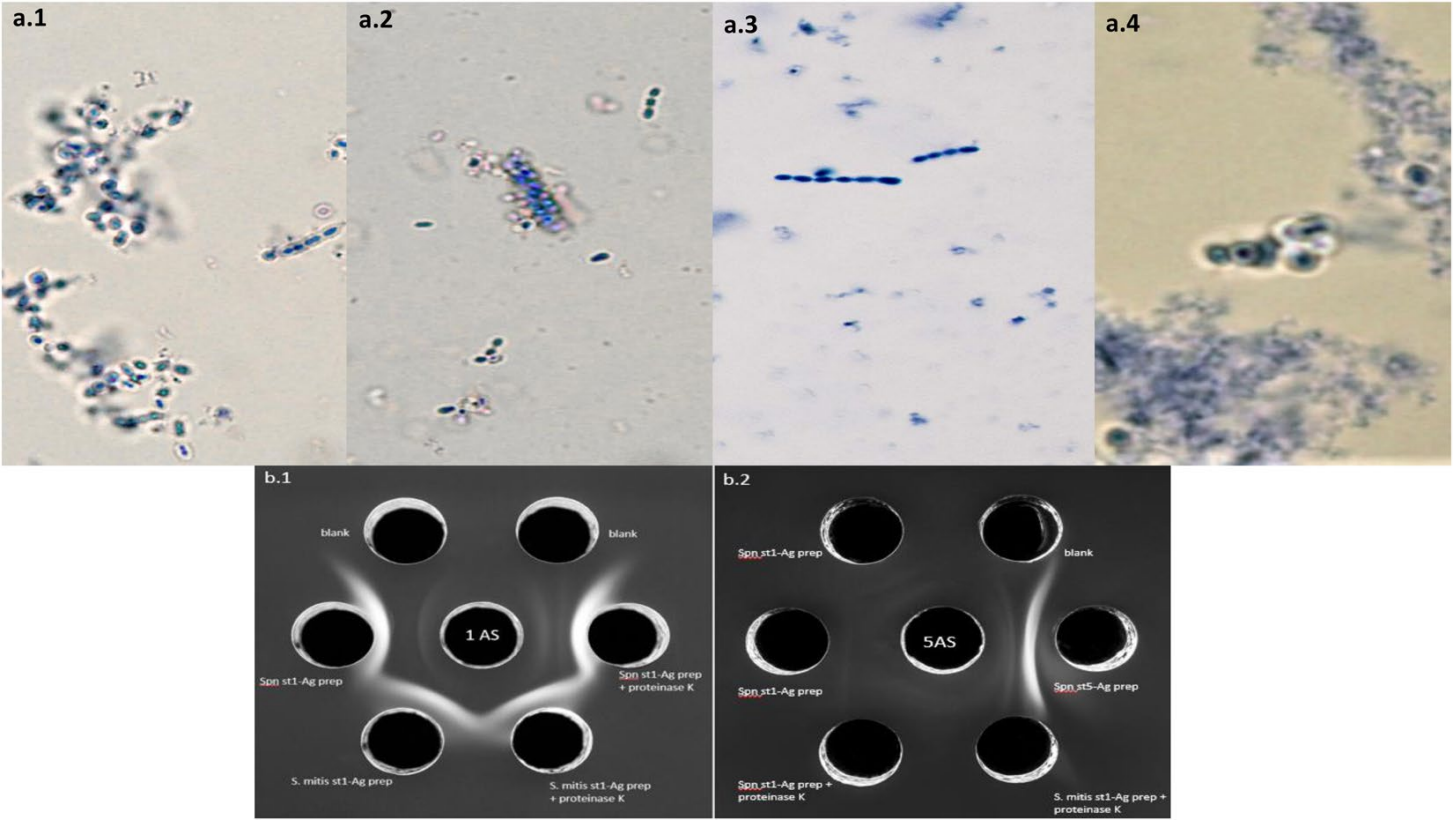
(**a**) Quellung reaction using *S*. *pneumoniae* serotyping 1 anti-serum for (**a**.**1**) *S*. *pneumoniae* serotype 1 strain (positive control), (**a**.**2**) *S*. *mitis* serotype 1 (L121) strain, and (**a**.**3**) *S*. *mitis* non-serotype 1 (NCTC12261-negative control) strain – no capsule visualization; and Quellung reaction using *S*. *mitis* serotyping 1 rabbit anti-serum obtained from L121 strain for (**a**.**4**) *S*. *pneumoniae* serotype 1 strain. (**b**) Double immunodiffusion assay testing (**b**.**1**) cross-reactive of serotype 1 *S*. *mitis* and *S*. *pneumoniae* capsule extracts with serotype 1 *S*. *pneumoniae* typing anti-serum, and (**b**.**2**) control reaction using serotype 1 *S*. *mitis* and serotype 1 and non-serotype 1 *S*. *pneumoniae* extracts with serotype 5 *S*. *pneumoniae* typing anti-serum. Blank – wells with no bacteria; 1AS – serotype 1 *S*. *pneumoniae* anti-serum; Spn st1 – *S*. *pneumoniae* serotype 1; 5AS – serotype 5 *S*. *pneumoniae* anti-serum; Spn st5 – *S*. *pneumoniae* serotype 5 (*S*. *mitis* serotype 1 L006 strain used in reaction).

**Figure 4:**
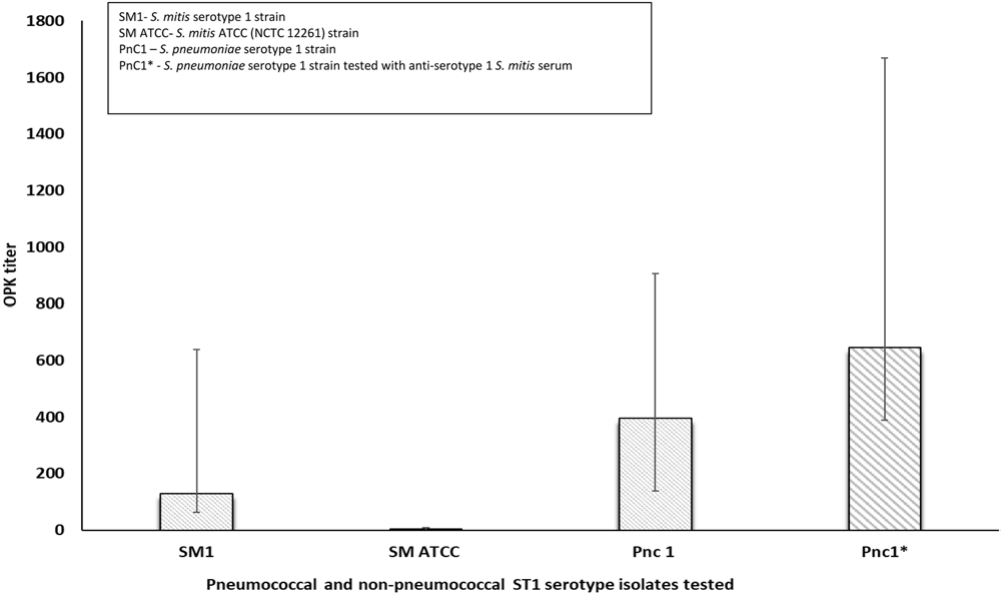
Opsonophagocytosis killing (OPK) testing of *S*. *mitis* and serotype 1 *S*. *pneumoniae* isolates using anti-serotype 1 pneumococcal and non-pneumococcal serum. Serotype 1 *S*. *pneumoniae* = positive control; *S*. *mitis* ATCC (NCTC 12261) strain confirmed to be *wzy1*-negative = negative control. Antibody source for OPK assay: 007 *S*. *pneumoniae* human reference serum. Complement: baby rabbit complement; OPK titer: reciprocal of serum dilution with >50% growth compared to serum free complement control. (*S*. *mitis* strain L006 and L121 representing two distinct *S*. *mitis* clones tested). Error bars represent the lower and upper limit of OPK titer based on the test runs.

Although subtle, pneumococcal serotype 1 capsule was visualized by Quellung reaction in three of the five *S*. *mitis* strains (L006, L121, L164; Table 2, Fig. 3a). Strains L006 and L121 representing two of the three *S*. *mitis* clones were tested using double immunodiffusion assays and showed strong cross-reactivity with *S*. *pneumoniae* serotype 1 typing anti-serum. Figure 3b shows side-by-side *S*. *mitis* serotype 1 antigen and *S*. *pneumoniae* serotype 1 antigen that generated symmetrical and fused precipitates in reaction with serotype 1 typing *S*. *pneumoniae* antisera. Based on an opsonophagocytosis killing (OPK) assay, capsule expressed by serotype 1 *S*. *mitis* and *S*. *pneumoniae* strains both triggered anti-serotype 1 antibody mediated opsonophagocytosis, while the *S*. *mitis* control strain (*wzy1*-negative) did not. In addition, opsonophagocytosis of serotype 1 pneumococci was observed with anti-serotype 1 *S*. *mitis* serum obtained from rabbits using L006 strain (Fig. 4).

## Discussion

In our large, 4-site study we found that few older US adults carried pneumococci, as detected either by culture or PCR for *lytA* on NP and OP specimens. We observed that, in spite of this lack of pneumococci, about 1 in 3 older adults had genes for known pneumococcal polysaccharide serotypes detected by PCR, and the gene for serotype 1 (*wzy1*) was the type most commonly detected. The finding that genes for PCV13 serotypes were significantly less common among adults who had received PCV13 suggested that the genes detected by PCR were likely linked to organisms that expressed capsules that cross reacted with antibodies generated by PCV13. Indeed, we found that *S*. *mitis* identified from 5 *wzy1*-positive specimens expressed capsules that were nearly identical to pneumococcal serotype 1 capsules by a range of common detection assays, genetic sequencing of the *cps* locus, and serologic assays.

Over 10% of the adults without pneumococcus detected within their upper respiratory tracts screened positive for the *wzy1*. This observation, combined with the characterization of the three distinct serotype 1 clones found in our study, suggests that a wide diversity of serotype 1 *S*. *mitis* strains may circulate within the adult US population. In our study population of adults aged over 64 years, carriage of *wzy1*-positive non-pneumococcal streptococci appeared to be more common than overall pneumococcal carriage. *S*. *mitis* colonization of the upper respiratory tracts persists from early infancy through adulthood, while *S*. *pneumoniae* colonization declines significantly with age^27^. In contrast to *S*. *pneumoniae*, *S*. *mitis* is only occasionally associated with disease^28^. Because the polysaccharide capsule is thought to be an important virulence factor for pneumococci, whether *S*. *mitis* that cause disease are typically those expressing a capsule should be explored. Other recent studies have shown that many *S*. *mitis* strains express a polysaccharide capsule, and animal models demonstrated that the capsule has a protective function in the clearance of *S*. *mitis*^23,29,30^. In addition, Engen *et al*.^31^ have recently identified similar and cross-reactive T helper cells (Th17) responses against commensal *S*. *mitis* and pathogenic *S*. *pneumoniae*. Exposure to *S*. *mitis* can induce T helper memory cells, which may lead to protection against pneumococcal carriage and disease^31^. The commensal *S*. *mitis* strains expressing a serotype 1 capsule that we found in healthy older US adults may induce a T cell response against serotype 1 *S*. *pneumoniae*, which in turn could theoretically provide protection against serotype 1 pneumococcal disease among colonized individuals. Our OPK assay results indicate that anti-serotype 1 pneumococcal human antibodies can clear *S*. *mitis* expressing serotype 1 capsule and that anti-serotype 1 *S*. *mitis* rabbit antibodies can clear serotype 1 pneumococci. If colonization with *S*. *mitis* serotype 1 strains induces an antibody response that can clear serotype 1 pneumococci, the notion that immunity induced by these relatively abundant and naturally occurring strains may explain the limited incidence of serotype 1 pneumococcal disease in the United States.

We found three distinct clones among the five *S*. *mitis* isolates. All three *S*. *mitis* clones shared *wzy1* operons that were highly similar to *wzy1* of the serotype 1 *S*. *pneumoniae* reference strain. We observed more genetic distance between the three *S*. *mitis* serotype 1 clones than among the 16 pneumococcal strains of different capsular serotypes, suggesting that *S*. *mitis* serotype 1 strain diversity is much higher than that observed between pneumococcal serotype 1 strains. Although some studies have suggested that capsular polysaccharides in *S*. *pneumoniae* evolved by importing relevant genes from a range of commensal *Streptococcus* species^23,32^, the *cps1* locus may have been transferred from pneumococcal strains to *S*. *mitis* strains, given the high level of background diversity found among *S*. *mitis* with serotype 1 capsule genes, or have been derived from a species that is no longer circulating. Therefore, predicting the directionality of serotype capsule genes transfers between *S*. *pneumoniae* and *S*. *mitis* is difficult.

Differences in the distribution of pneumococcal serotypes causing disease have been observed among populations^4^. However, more work is needed to understand if the distribution of capsular polysaccharide serotypes in commensal streptococci affect the pneumococcal serotypes causing disease. Cross-reactivity between encapsulated *S*. *mitis* and *S*. *pneumoniae* has recently been reported by the Danish group using immunodiffusion assays^23^. However, serotype 1 *S*. *mitis* were not observed among the 66 *S*. *mitis* isolates examined, which were mostly from Denmark (N = 42) and non-vaccine serotypes. In a Kenya carriage study, we were also unable to detect *wzy1*–positive non-pneumococcal Mitis group strains from NP or OP specimens from 158 adults^33^. In both Denmark and Kenya, serotype 1 is a common serotype causing IPD, while in the United States this serotype has been an uncommon cause of disease in recent decades^2,34,35^.

Among our study participants, pneumococci were rarely isolated from OP specimens, yet these specimens were much more likely than NP specimens to be positive by PCR for vaccine-serotype polysaccharide capsule genes; often such specimens were *lytA*-negative, providing evidence that the source of the serotype genes were not pneumococci. The oral cavity contains a great diversity of streptococcal species, and pneumococcal PCR serotyping assays can often identify homologs among mitis group non-pneumococcal strains^33^. Pneumococcal carriage studies using PCR-based detection of serotype-specific genes directly on OP specimens can overestimate vaccine type pneumococcal carriage rates given the high prevalence of confounding non-pneumococcal species carrying homologs of pneumococcal-serotype specific genes. In addition, the *lytA* gene for pneumococcal detection is not entirely specific. A recent study from Portugal identified a *lytA*-positive *S*. *pseudopneumoniae* strain in sputum^36^. In our study, two of 224 non-pneumococcal streptococci colonies screened for *lytA* were positive (data not shown). The use of PCR-based detection directly on upper respiratory specimens is of questionable value for pneumococcal carriage studies designed to measure vaccine impact based on the high prevalence of serotype 1 and other serotype genes on specimens that did not contain pneumococci. Although OP specimens had higher positivity for *wzy1*, 7 NP specimens that were *lytA*-negative were *wzy1*-positive by real-time PCR. Other studies have also shown problems with real-time PCR for serotyping of NP specimens^37^.

In conclusion, we found that genes for polysaccharide capsules similar to those expressed by pneumococci, in particular genes for serotype 1, were common among our sample of older US adults. The serotype 1 genes belonged to *S*. *mitis* strains that expressed capsules immunologically indistinguishable from pneumococcal capsules. Our findings raise many questions, in particular about the role of commensal streptococci in the development and maintenance of natural immunity to pneumococci. Further work should focus on these questions and whether the presence of capsule-containing non-pneumococcal streptococci affects the serotype distribution and amount of disease caused by pneumococci in different population. These strains could provide a novel approach for enhancing the human microbiome’s ability to protect against serotype 1 pneumococcal disease, which is responsible for 11.7% of all IPD cases in Africa^4^ and continue to be the main serotype causing pneumococcal meningitis outbreaks in the African meningitis belt countries that have already introduced pneumococcal conjugate vaccine^8^. Given the genetic similarities between capsular loci, those working with molecular assays to evaluate pneumococcal serotypes in the upper respiratory tract should consider whether non-pneumococcal streptococci strains with capsular genes may affect their results.

## Electronic supplementary material

Supplementary Information
