# Supplementary material for: Streptococcus mitis Expressing Pneumococcal Serotype 1 Capsule
Source: Sci Rep. 2018 Dec 19;8:17959. doi: 10.1038/s41598-018-35921-3 (PMC6299277; doi:10.1038/s41598-018-35921-3)

Streptococcus mitis Expressing Pneumococcal Serotype 1 Capsule

Fernanda C. Lessa, MD[1*]; Jennifer Milucky, MSPH[1]; Nadine Rouphael, MD[2]; Nancy M. Bennett, MD[3]; H. Keipp Talbot, MD[4]; Lee H. Harrison, MD[5], Monica M. Farley, MD[2,6] , Jeremy Walston, MD[7], Fabiana Pimenta, PhD[1], Robert E. Gertz, MS[1], Gowrisankar Rajam, PhD[1]; Maria da Gloria Carvalho, PhD[1]; Bernard Beall, PhD[1*]; Cynthia G. Whitney, MD[1]

**Corresponding authors*

Affiliations:

[1]Centers for Disease Control and Prevention, National Center for Immunization and Respiratory Diseases, Division of Bacterial Diseases, Atlanta, Georgia

[2]Emory University School of Medicine, Department of Medicine, Atlanta, Georgia

[3]University of Rochester School of Medicine and Dentistry, Department of Medicine, Rochester, New York.

[4]Vanderbilt University Medical Center, Nashville, Tennessee.

[5]Johns Hopkins Bloomberg School of Public Health, Baltimore, Maryland.

[6] Atlanta Veterans Affairs Medical Center, Atlanta, Georgia.

[7] Division of Geriatric Medicine and Gerontology, Johns Hopkins University School of Medicine.

Corresponding authors: Fernanda C. Lessa (flessa@cdc.gov, 404-639-7798) and Bernard Beall (bbeall@cdc.gov, 404-639-1237), 1600 Clifton Road, MS A-24, Atlanta, GA 30333

SUPPLEMENTARY MATERIAL

Supplementary Figure: Detection of Pneumococcus by Culture or PCR on Upper Respiratory Tract Specimens from US Adults, July 2015-March2016

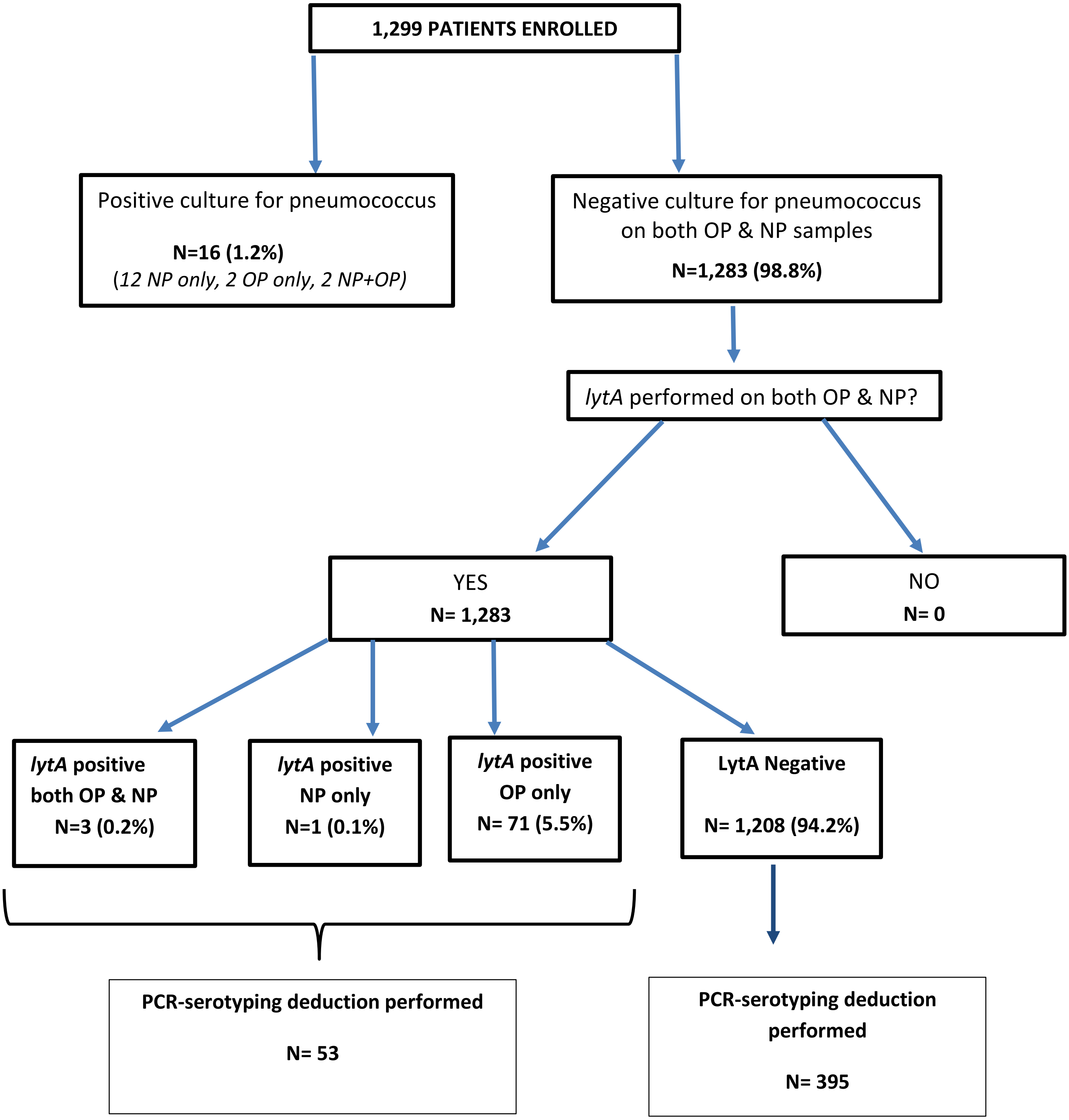

Supplement: Supplementary file 1 — Supplementary Information [file 41598_2018_35921_MOESM1_ESM.pdf]
